# Predicting late-stage age-related macular degeneration by integrating marginally weak SNPs in GWA studies

**DOI:** 10.3389/fgene.2023.1075824

**Published:** 2023-03-30

**Authors:** Xueping Zhou, Jipeng Zhang, Ying Ding, Heng Huang, Yanming Li, Wei Chen

**Affiliations:** ^1^ Department of Biostatistics, University of Pittsburgh, Pittsburgh, PA, United States; ^2^ Department of Electrical and Computer Engineering, University of Pittsburgh, Pittsburgh, PA, United States; ^3^ Department of Biostatistics and Data Science, University of Kansas Medical Center, Kansas, KS, United States; ^4^ Department of Pediatrics, University of Pittsburgh, Pittsburgh, PA, United States

**Keywords:** age-related macular degeneration, biomarker discovery, feature selection, genome-wide association study, linkage disequilibrium, prediction, weak signal

## Abstract

**Introduction:** Age-related macular degeneration (AMD) is a progressive neurodegenerative disease and the leading cause of blindness in developed countries. Current genome-wide association studies (GWAS) for late-stage age-related macular degeneration are mainly single-marker-based approaches, which investigate one Single-Nucleotide Polymorphism (SNP) at a time and postpone the integration of inter-marker Linkage-disequilibrium (LD) information in the downstream fine mappings. Recent studies showed that directly incorporating inter-marker connection/correlation into variants detection can help discover novel marginally weak single-nucleotide polymorphisms, which are often missed in conventional genome-wide association studies, and can also help improve disease prediction accuracy.

**Methods:** Single-marker analysis is performed first to detect marginally strong single-nucleotide polymorphisms. Then the whole-genome linkage-disequilibrium spectrum is explored and used to search for high-linkage-disequilibrium connected single-nucleotide polymorphism clusters for each strong single-nucleotide polymorphism detected. Marginally weak single-nucleotide polymorphisms are selected *via* a joint linear discriminant model with the detected single-nucleotide polymorphism clusters. Prediction is made based on the selected strong and weak single-nucleotide polymorphisms.

**Results:** Several previously identified late-stage age-related macular degeneration susceptibility genes, for example, *BTBD16*, *C3*, *CFH*, *CFHR3*, *HTARA1*, are confirmed. Novel genes *DENND1B*, *PLK5*, *ARHGAP45*, and *BAG6* are discovered as marginally weak signals. Overall prediction accuracy of 76.8% and 73.2% was achieved with and without the inclusion of the identified marginally weak signals, respectively.

**Conclusion:** Marginally weak single-nucleotide polymorphisms, detected from integrating inter-marker linkage-disequilibrium information, may have strong predictive effects on age-related macular degeneration. Detecting and integrating such marginally weak signals can help with a better understanding of the underlying disease-development mechanisms for age-related macular degeneration and more accurate prognostics.

## 1 Introduction

AMD is a progressive neurodegenerative disease characterized by reduced retinal pigment epithelium (RPE) function and loss of photoreceptors in the macula (Fritsche et al., 2016). By 2040, there will likely be 288 million AMD patients worldwide (Wong et al., 2014). Between 6 and 9 percent of blindness worldwide is caused by AMD, which is also the leading cause of blindness in developed countries (Wong et al., 2014; Jonas et al., 2017; Keenan et al., 2021). AMD is divided into early, intermediate, and late stages. In the first two stages, the visual symptoms are typically absent or mild. Late-stage AMD involves two forms: geographic atrophy (GA) and choroidal neovascularization (CNV), though the two manifestations are not mutually exclusive (Keenan et al., 2021; Ung et al., 2021). Either form of late AMD can lead to vision distortion and/or vision loss (Wong et al., 2014; Keenan et al., 2021). Until now, understanding of the disease biology and therapeutic options are limited (Fritsche et al., 2014; Fritsche et al., 2016). Age, genetics, and environmental factors are all expected to cause the disease in a complex interactive way (Keenan et al., 2021). Previous research has identified susceptibility SNPs and genes for the development of late-stage AMD (Fritsche et al., 2013; Kvale et al., 2015; Fritsche et al., 2016). However, the current identified genetic biomarkers only explained a small portion of the disease variability. It would be extremely helpful to identify novel biomarkers pertinent to late-stage AMD for a better understanding of the disease etiology and for developing new tools to aid accurate prognostic and precision treatment.

A recent landmark genome-wide association study (GWAS) has identified 52 independently associated variants across 34 susceptibility loci for AMD by comparing the late-stage AMD patients and unaffected controls of European ancestry (Fritsche et al., 2016). Even though GWAS is a very effective method for identifying SNPs associated with diseases, the majority of GWAS research selects significant biomarkers based on single marker approaches, where each SNP is evaluated separately for its marginal correlation with the outcome. As a result, the correlation spectrum between SNPs across the genome is ignored, and only SNPs with strong marginal effects can be selected. In Fritsche et al. (2016), the 52 identified variants explained only 27.2% of the disease variability. Recent studies have shown that incorporating inter-feature correlations/connections can help identify novel genetic biomarkers and improve outcome prediction accuracy (Li, 2019; Li et al., 2019a; Li et al., 2019b). This suggests that we might be able to discover new AMD variants and explain more disease variability by exploring and integrating the genome-wide correlation/connectivity patterns into the GWAS. Following Li (2019); Li et al. (2019b), we term the variants that cannot be directly detected but would exhibit significant differentiating effects after taking into consideration of their correlations/connections to other genome-wide significant SNPs as the marginally weak SNPs, or weak SNPs. In this paper, we aim to detect weak SNPs for late-stage AMD, investigate their biological function, and confirm whether they can help to improve disease prediction.

We use linkage disequilibrium (LD) as the metric to measure the inter-SNP correlations. In genetics, “LD” refers to the non-random correlations between alleles at different loci (Slatkin, 2008). It has been used to determine correlated disease-related SNP sets and is a crucial metric for determining inter-SNP interaction (Carlson et al., 2004). The squared correlation, or *r* ^2^ statistic, which spans from 0 to 1, is one of the most widely used LD metrics. A higher *r* ^2^ value indicates a stronger correlation between two SNPs. In this study, we use *r* ^2^ to explore the whole-genome LD spectrum. For each genomewide significant SNP identified from a GWA scan, we detect its high-LD connected SNP cluster using connected component searching algorithms (Li et al., 2019b; Li, 2019), such that any pair of SNPs within the same cluster are connected through a path of high LD links. A marginally weak SNP can be then selected based on not only its own marginally differentiating effect but also how many other SNPs within the cluster it connects to and the disease-differentiating capabilities of those connected SNPs (Li et al., 2019b).

## 2 Materials and methods

### 2.1 Data

International Age-Related Macular Degeneration Genomics Consortium (IAMDGC) has collected genetic data from AMD patients and control subjects across 26 different studies. IAMDGC dataset is by far the largest-scaled AMD GWAS dataset (Fritsche et al., 2016). In this study, we used a total of 27,301 unrelated individuals of European ancestry in the IAMDGC dataset, including 14,590 late-stage AMD patients with GA and/or CNV and 12,711 normal controls. Genotypes and phenotypes for these 27,301 samples were downloaded from NCBI dbGap database.

Our analysis included a total of 5,002,684 common SNPs from chromosomes 1 through 22 with minor allele frequency (MAF) larger than 0.1. Each SNP is coded by its number of minor alleles (0, 1, or 2). It is known from previous studies that this sample population does not contain an obvious heterogeneous structure. We also carried out a principal component analysis (PCA). The PCA results showed no obvious population structures. We calculated the kinship matrix using 100,000 randomly sampled SNPs across the whole genome. The absolute values of the KING-robust estimators on 100,000 randomly chosen sub-principal matrices were very small (mean = 0.0077, standard deviation = 0.0081). All the above evidence suggests that the samples used in our study are independent. Following previous studies using the same data cohort (Fritsche et al., 2016), we did not adjust for PC scores in our study. The missing genotype values were imputed by the means of the non-missing ones. There are 2.1% of the participants older than 90 years. We capped their ages at 90 in the analysis. There are 0.4% of the samples with missing ages and their ages were imputed by the average age of non-missing subjects.

## 3 Methods

### 3.1 Notation

Denote the observed data matrix by ***X***_*n*×*p*_, where *n* is the number of subjects and *p* is the total number of features. We use *X*_*j*_ to denote the length-*n* sample vector for feature *j*, *j* = 1, … , *p*. Let **y** denotes the length-*n* vector of outcome class labels, and *y*_*i*_ denotes the class indicator for subject *i*, *i* = 1, … , *n*. We use *n*_*k*_ to denote the size of class *k*, *k* = 0, 1, where *k* = 0 for normal controls and *k* = 1 for late-stage AMD patients.

### 3.2 Detection of marginally strong SNPs

Marginal Student t-tests were used to select marginally strong signals. Specifically, for each SNP *j*, *j* = 1, … , *p*, we calculated

$${\hat{T}}_{j}=\left({\bar{X}}_{j}^{\left[1\right]}-{\bar{X}}_{j}^{\left[0\right]}\right)/s_{\Delta },$$ (1)

where ${\bar{X}}_{j}^{\left[1\right]}=\frac{\sum_{Y_{i}=1}X_{i,j}}{n_{1}}$ , ${\bar{X}}_{j}^{\left[0\right]}=\frac{\sum_{Y_{i}=0}X_{i,j}}{n_{0}}$ and $s_{\Delta }=\sqrt{\sum \sum {(X_{i,j}-{\bar{X}}_{j}^{\left[k\right]})}^{2}/\left(n_{0}+n_{1}\right)}$ . SNPs with *p*-values less than 5 × 10^−8^ were selected as the marginally strong SNPs.

### 3.3 Detection of high-LD connected SNP clusters

We used PLINK 1.9 (Purcell et al., 2007), a tool set for whole-genome association and population-based linkage analyses, to calculate *r*2 values for all SNPs on each chromosome. We then thresholded *r* ^2^ values at 0.7 so that only SNPs in high LD were considered connected and included in the downstream analyses. For each marginally strong SNP, we employed the connected component searching algorithms in Li (2019); Li et al. (2019b) to detect the cluster of SNPs each of which is connected to the strong SNP through a path of high-LD links. Suppose there are *B* such clusters detected, we denote those clusters by *C*_*l*_, where *l* = 1, … , *B*.

### 3.4 Detection of marginally weak SNPs

LD-adjusted effect sizes were then calculated for SNPs in the detected connected components. Specifically, for each *C*_*l*_, *l* = 1, … , *B*, we calculated the multivariate t-statistic by ${\hat{T}}_{C_{l}}^{ld}={\hat{\Omega }}_{C_{l}}\left({\bar{X}}_{j}^{\left[1\right]}-{\bar{X}}_{j}^{\left[0\right]}\right)$ , where ${\hat{\Omega }}_{C_{l}}={\hat{\Sigma }}_{C_{l}}^{-1}$ is the inverse of the LD matrix ${\hat{\Sigma }}_{C_{l}}$ on component *C*_*l*_. ${\hat{\Omega }}_{C_{l}}$ characterizes the LD connections between SNPs in *C*_*l*_. For each SNP *j* in *C*_*l*_, its LD-adjusted differentiating statistic is the corresponding entry in ${\hat{T}}_{C_{l}}^{ld}$

$${{\hat{T}}_{j}}^{ld}=\underset{j^{\prime }\in c_{l}}{\sum }{\left[{\hat{\Omega }}_{C_{l}}\right]}_{jj^{\prime }}\left({\bar{X}}_{j}^{\left[1\right]}-{\bar{X}}_{j}^{\left[0\right]}\right).$$ (2)

The greater the magnitude of ${{\hat{T}}_{j}}^{ld}$ is, the higher power SNP *j* has in differentiating the diseases and controls after integrating the LD information with its connected SNPs. Marginally weak SNPs were selected based on $\left|{{\hat{T}}_{j}}^{ld}\right|$ . It should be noted that the calculation of the connection matrix Ω is essential for marginally weak signal detection (Li et al., 2019b). For an ultra-high dimensional association study like a GWAS, it is impossible to directly calculate $\hat{\Omega }$ for all SNPs. By leveraging the marginal LD information, we were able to break it down to locally connected components *C*_*l*_s, which were of much smaller sizes, thus making the calculation feasible. Not every SNP within an LD-connected cluster would necessarily be informative for classification. Only the top 1,000 SNPs with the greatest $\left|{\hat{T}}_{j}^{ld}\right|$ values, aside of the strong SNPs, were selected as the marginally weak SNPs. The optimal hyperparameter value of 1,000 was chosen by a cross-validation procedure.

### 3.5 Calculating *p*-values for selected SNPs

To evaluate the statistical significance of the selected SNPs, a post-selection non-parametric permutation test was performed. We first constructed a permutation dataset by re-sampling (with replacement) the class labels but keeping the genotype matrix unchanged. We kept the detected strong SNP set and the connected components unchanged to preserve the connection patterns. We then recalculated the statistics $T_{j}^{ld}$ using the original ${\hat{\Omega }}_{C_{l}}$ , and ${\bar{X}}_{j}^{\left[1\right]}$ and ${\bar{X}}_{j}^{\left[0\right]}$ recalculated from the permuted dataset. An empirical null distribution of ${\hat{T}}_{j}^{cl,\left(m\right)}$ , *m* = 1, … , *M*, was then generated by repeating the process *M* = 120,000 times. The percentage of the occurrences that the permuted statistics $\left|{\hat{T}}_{j}^{cl,\left(m\right)}\right|$ were equal to or more extreme than the observed statistics $\left|{{\hat{T}}_{j}}^{ld}\right|$ (evaluated from the original non-permuted dataset) was defined to be the permutation test *p*-value for each selected SNP *j*. Last, to further control for the family-wise error rate, a Bonferroni correction was made for all selected SNPs.

### 3.6 Functional enrichment analysis

All SNPs in the analysis were first mapped onto genes in the Human Genome GRCh37/hg19 assembly using the R package “biomaRt” (Durinck et al., 2009). A SNP not in any gene region was mapped to its closest nearby gene containing it within its 2 kb upstream/downstream ranges. Functional enrichment analysis was then performed to validate the association of the selected SNPs with AMD from a system biology perspective. Ingenuity Pathway Analysis (IPA, QIAGEN, Inc.) was used to identify the significantly enriched canonical pathways. Fisher’s exact test *p*-values were provided for each canonical pathway. A pathway with a *p*-value less than 0.01 was considered a significantly enriched pathway.

For each significantly enriched pathway, we reported its leading-edge genes. Leading edge genes can be viewed as the pivot genes in the pathway because of which the pathway got enriched (Subramanian et al., 2005). We evaluated how the leading-edge genes of the enriched pathways overlapped with selected genes. A greater overlap indicates more compelling evidence of the biological functions of the selected genes/SNPs on AMD.

### 3.7 Late-stage AMD prediction

We fitted a logistic regression with a Lasso penalty and the selected marginally strong and weak SNPs for outcome prediction. We included age and gender as adjusting covariate variables.

The whole analysis pipeline is summarized in Algorithm 1.

Algorithm 1 Procedure for detecting strong and weak signals and late-stage AMD classification.

1: **Marginally strong SNPs:** From marginal *t*-tests.
2: **LD connected SNP clusters:** For each strong SNP, detecting its high-LD connected SNP clustered by searching for its connected component in the thresholded LD matrix.
3: **Marginally weak SNPs:** Detected by ranking the LD-adjusted statistics $\left|{\hat{T}}_{C_{l}}^{ld}|=|{\hat{\Omega }}_{C_{l}}\left({\bar{X}}_{j}^{\left[1\right]}-{\bar{X}}_{j}^{\left[0\right]}\right)\right|$ .
4: **Functional enrichment analysis**.
5: **Late-stage AMD prediction:** Prediction using Lasso-logistic regression with selected SNPs and adjusting covariates.

## 4 Results

### 4.1 Selected candidate SNPs and associate genes

**FIGURE 1:**
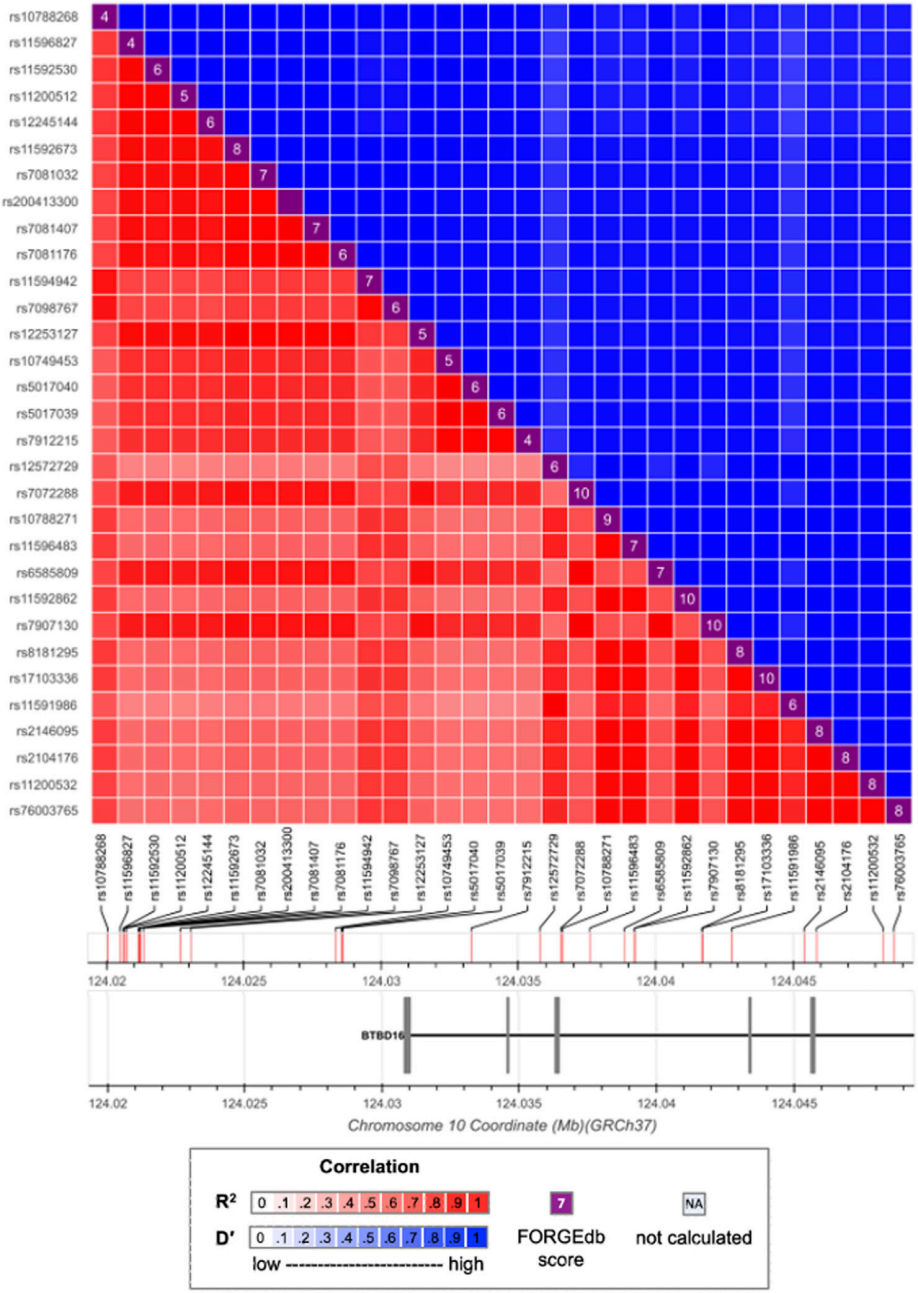
LD pattern between strong and weak SNPs. Marginally strong SNPs: rs57581057, rs10788268, rs11594942, rs7098767, rs12572729, rs10788271, rs11596483, rs8181295, rs17103336, rs11591986, rs2146095, rs2104176, rs11200532, rs76003765. Marginally weak SNP: rs790713.

Variants selection was conducted on each of the 22 autosomes separately. There were 4,679 genome-wide significant SNPs detected as the marginally strong SNPs, of which 26 had been reported by Fritsche et al. (2016). We further detected 3,446 high-LD connected SNPs, each of which had an *r* ^2^ > 0.7 with at least one of the marginally strong SNPs. Those 3,446 SNPs were clustered within 410 connected components. Figure 1 shows an example connected component of 32 SNPs, out of which 14 were marginally strong SNPs and 1 was eventually selected as marginally weak SNPs. After the final filtering, the top 1,000 SNPs out of those 3,446 with the greatest $\left|{\hat{T}}_{C_{l}}^{ld}\right|$ values were selected as the marginally weak SNPs. Altogether, there were 5,679 SNPs (4,679 marginally strong and 1,000 marginally weak) selected as the informative SNPs. Those 5,679 SNPs were then mapped to 197 gene regions, with 99 genes mapped by marginally strong SNPs only, 35 genes mapped by marginally weak SNPs only, and 63 genes mapped by both marginally strong and weak SNPs (Supplementary Table S1).

In Figure 1, the marginally weak SNP rs7907130 has a marginal *p*-value = 1.124 × 10^−4^. As a result, it is likely to be missed from downstream fine mappings in a conventional GWAS. However, Once the LD information is integrated, SNP rs7907130 has the most significant permutation test *p*-value among all the 32 SNPs. Therefore, its predictive effect is manifested through integrating its LD connections with the strong SNPs. Moreover, SNP rs7907130 was mapped to a previously identified gene *BTBD16* (Strunz et al., 2020a) together with the other strong SNPs. It could potentially be a new AMD susceptibility locus.

### 4.2 Statistically significance for selected SNPs and genes

Post-selection inference was made for the 5,679 SNPs selected. A Bonferroni correction gave a family-wise significance level of 8.805 × 10^−6^ (= 0.05/5, 679). Based on the permutation test results, 55 out of the 4,679 selected marginally strong SNPs (Table 1 and the full list in Supplementary Table 4) and 13 out of the 1,000 selected marginally weak SNPs (Table 2) were statistically significant with a permutation *p*-value $<8.805\times 10^{-6}$ . Table 1 gives the detailed test results for the top marginally strong SNPs and their mapped genes. Table 2 gives the detailed test results for the significant marginally-weak SNPs and their mapped genes.

**TABLE 1:** Permutation *p*-values of selected marginally strong SNPs and their mapped genes.

SNP	Chr:pos	Gene	*p*-value	AMD-related literature for gene
rs6695321	1:196675861	*CFH*	$<8.805\times 10^{-6}$	Guenther et al. (2020), Tian et al. (2012) ^†^
rs7524776	1:196623337	*CFH*	$<8.805\times 10^{-6}$	Budzinskaia et al. (2011) ^†^
rs201084593	1:197316970	*CRB1*	$<8.805\times 10^{-6}$	Fletcher et al. (2014)
rs4915551	1:197508901	*DENND1B*	$<8.805\times 10^{-6}$	
rs10429910	1:197141253	*ZBTB41*	$<8.805\times 10^{-6}$	Zhang et al. (2008)
rs4865172	4:57907587	*IGFBP7*	$<8.805\times 10^{-6}$	Arakawa et al. (2011)
rs3132451	6:31582025	*AIF1*	$<8.805\times 10^{-6}$	Wolf et al. (2022)
rs201943238	6:32314956	*TSBP1, TSBP1-AS1*	$<8.805\times 10^{-6}$	Strunz (2021)
rs1043618	6:31783507	*HSPA1A*	$<8.805\times 10^{-6}$	Liang et al. (2022)
rs444921	6:31932177	*SKIV2L*	$<8.805\times 10^{-6}$	Kopplin et al. (2010)
rs406658	6:31996524	*C4B*	$<8.805\times 10^{-6}$	Grassmann et al. (2016)
rs6906021	6:32626311	*HLA-DQB1-AS1*	$<8.805\times 10^{-6}$	Jorgenson et al. (2016)
rs5788544	10:124091856	*BTBD16*	$<8.805\times 10^{-6}$	Strunz et al. (2020a)
rs2300431	10:124242817	*HTRA1*	$<8.805\times 10^{-6}$	Gibbs et al. (2008)^†^
rs7093894	10:124234880	*HTRA1*	$<8.805\times 10^{-6}$	Goto et al. (2009)^†^
rs2239586	10:124249235	*HTRA1*	$<8.805\times 10^{-6}$	Tam et al. (2008)^†^
rs6857	19:45392254	*NECTIN2/PVRL2*	$<8.805\times 10^{-6}$	Holliday et al. (2013)^†^
rs265285	19:1534676	*PLK5*	$<8.805\times 10^{-6}$	
rs265284	19:1534685	*PLK5*	$<8.805\times 10^{-6}$	
rs2074453	19:1080189	*ARHGAP45/HMHA1*	$<8.805\times 10^{-6}$	

• Chr:pos—chromosome and position.

• †—the SNP is also previously reported in the study.

**TABLE 2:** Permutation *p*-values of selected marginally weak SNPs and their mapped genes.

SNP	*p*-value	Chr:pos	Gene	Gege literature	Strong SNP in high LD
rs10489456	$<8.805\times 10^{-6}$	1:196687515	*CFH*	Ennis et al. (2007)^†^	rs388419 (1:196747669)
					rs449657 (1:196747834)
					rs201334308 (1:196747111)
rs72468003	$<8.805\times 10^{-6}$	1:196883372	*CFHR4*	Kubista et al. (2011)	rs114092527 (1:196894694)
rs10442656	$<8.805\times 10^{-6}$	1:197606708	*DENND1B*		rs4915551 (1:197508901)
rs13150593	$<8.805\times 10^{-6}$	4:57810321	None		rs4865172 (4:57907587)
rs17197637	7.311 × 10^−6^	6:31210502	None		rs112340183 (6:31228410)
rs2844603	$<8.805\times 10^{-6}$	6:31250854	None		rs112340183 (6:31228410)
rs2853931	$<8.805\times 10^{-6}$	6:31255007	None		rs112340183 (6:31228410)
rs2242655	$<8.805\times 10^{-6}$	6:31627449	*C6orf47, BAG6*		rs112340183 (6:31228410)
rs805304	$<8.805\times 10^{-6}$	6:31698088	*DDAH2*	Lange et al. (2016)	rs1043618 (6:31783507)
			*CLIC1*	Chuang et al. (2022)	rs1043618 (6:31783507)
rs72887130	$<8.805\times 10^{-6}$	6:50902701	None		rs3857599 (6:50938247)
rs16880854	$<8.805\times 10^{-6}$	6:50904881	None		rs3857599 (6:50938247)
rs3857596	$<8.805\times 10^{-6}$	6:50905067	None		rs3857599 (6:50938247)
rs7907130	$<8.805\times 10^{-6}$	10:124039235	*BTBD16*	Strunz et al. (2020a)	rs7098767 (10:124022694)
rs9747347	$<8.805\times 10^{-6}$	17:79606820	*TSPAN10*	Strunz et al. (2020b)	rs6565597 (17:79526821)

• Chr:pos—chromosome and position.

• †—the SNP is also previously reported in the study.

The majority of the detected SNPs and their mapped genes had been reported to be associated with AMD previously. However, five new genes have been identified in our study as the potentially novel susceptibility genes for late-stage AMD: *DENND1B*, *PLK5*, *ARHGAP45/HMHA1*, *C6orf47*, and *BAG6*.

*DENND1B* (differentially expressed in normal versus neoplastic domain containing 1B) is mapped by one marginally strong and one marginally weak SNP. Chronic inflammatory illnesses like asthma (Sleiman et al., 2010), inflammatory bowel disease (Lees et al., 2011), and primary biliary cirrhosis (Mells et al., 2011) have been reported associated with this gene. The disease pathogenesis involves the *DENND1B* encoded protein and its interaction with tumor necrosis factor *α* (TNF-*α*) (Sleiman et al., 2010). Although *DENND1B* has not been directly linked to AMD, it may play a similar role in AMD pathogenesis through inflammation related to TNF-*α*, which is a pro-inflammatory cytokine contributing to inflammation-associated angiogenesis and CNV (Jasielska et al., 2010; Khan et al., 2021). *ARHGAP45* (rho GTPase activating protein 45) or *HMHA1* (minor histocompatibility antigen 1) is mapped by one marginally strong SNP. It controls endothelial integrity, whereas endothelial barrier dysfunction can lead to uncontrolled leaks or edema (Amado-Azevedo et al., 2018). In CNV, aberrant blood vessels leak fluid or blood into the retina. *ARHGAP45* may contribute to AMD by regulating the endothelial barrier function of blood vessels. *PLK5* (polo-like kinase 5) is associated with two marginally strong SNPs. It can induce genotoxic stress and DNA damage (Andrysik et al., 2010), which is one of the underlying mechanisms of AMD. Additionally, *PLK5* is specifically expressed in the eye (de Cárcer et al., 2011) and is a candidate gene modulating the proper development and function of mice eyes (Chiang et al., 2020).

*BAG6* (BAG Cochaperone 6) and *C6orf47* (chromosome 6 open reading frame 47) are mapped by one significant marginally weak SNP. *BAG6* gene is found to be a component of a cluster of immune-relevant genes located within the human major histocompatibility complex class (MHC) III region (Banerji et al., 1990). *BAG6* encodes a multifunctional protein. For instance, the protein is required for the acetylation of p53 in response to DNA damage (Binici and Koch, 2014), which is believed to be involved in the RPE cell death in AMD (Bhattacharya et al., 2012; Vuong et al., 2012). *C6orf47* gene has been reported related to inflammatory diseases (Harney et al., 2008; Goudey et al., 2017). However, its biological functions remain unclear (Ren et al., 2015) and require further investigation.

### 4.3 Functional enrichment analyses

We further conducted two functional enrichment analyses to verify the biological functions of the selected genes, especially the 35 genes mapped from marginally weak SNPs. We examined two candidate gene lists. One list consists of all the 197 genes mapped by both marginally strong and marginally weak SNPs. The other list consists of 162 genes mapped from marginally strong SNPs only. IPA software was used for the enrichment analyses. Any differences between the results from the two enrichment analyses should be due to the marginally weak genes and can help reveal the biological significance of these weak genes. Table 3 summarizes all enriched pathways identified by the two gene lists with a *p*-value of <0.01 . Nine canonical pathways were significantly enriched by both candidate gene lists. Another nine canonical pathways were significantly enriched only when marginally weak genes were included.

**TABLE 3:** Enriched canonical pathways from Ingenuity Pathway Analysis (IPA).

Ingenuity canonical pathway	Ratio	Pathway genes	*p*-value
Complement System	0.162	*C2*, *C3*, *C4A/C4B*, *CFD*, *CFH*, *CFI*	2.63 × 10^−7^
LXR/RXR Activation	0.073	*ABCA1*, *APOC1*, *APOE*, *APOM*, *C3*, *C4A/C4B*, *CETP*	3.09 × 10^−7^
		***MMP9***, ***VTN***	
Antigen Presentation Pathway	0.154	*HLA-B*, *HLA-DQA1*, *HLA-DQB1*	3.72 × 10^−7^
		***HLA-A***, ***HLA-C***, ***HLA-DRA***	
Neuroprotective Role of *THOP1*	0.067	*CFD*, *HLA-A*, *HTRA1*, *PRSS57*, *PRTN3*	2.88 × 10^−6^
in Alzheimer’s Disease		***HLA-B***, ***HLA-C***, ***MMP9***	
FXR/RXR Activation	0.064	*APOC1*, *APOE*, *APOM*, *C3*, *C4A/C4B*, *CETP*, *LIPC*, ***VTN***	4.07 × 10^−6^
PD-1, PD-L1 cancer immunotherapy*	0.066	*HLA-B*, *HLA-DQA1*, *HLA-DQB1*	1.29 × 10^−5^
		***CSNK2B***, ***HLA-A***, ***HLA-C***, ***HLA-DRA***	
B Cell Development	0.114	*HLA-B*, *HLA-DQA1*, *HLA-DQB1*	1.70 × 10^−5^
		***HLA-A***, ***HLA-DRA***	
Neuroinflammation Signaling Pathway*	0.035	*AGER*, *GRIN3B*, *HLA-B*, *HLA-DQA1*, *HLA-DQB1*, *PLA2G12A*	2.24 × 10^−5^
		***HLA-A***, ***HLA-C***, ***HLA-DRA***, ***MMP9***, ***TGFBR1***	
Crosstalk between Dendritic Cells and	0.066	HLA-B, MICB, NECTIN2	5.50 × 10^−5^
Natural Killer Cells*		***HLA-A***, ***HLA-C***, ***HLA-DRA***	
Th2 Pathway	0.051	*HLA-B*, *HLA-DQA1*, *HLA-DQB1*, *NOTCH4*	6.76 × 10^−5^
		***HLA-A***, ***HLA-DRA***, ***TGFBR1***	
Th1 and Th2 Activation Pathway*	0.041	*HLA-B*, *HLA-DQA1*, *HLA-DQB1*, *NOTCH4*	2.75 × 10^−4^
		***HLA-A***, ***HLA-DRA***, ***TGFBR1***	
Th1 Pathway	0.049	*HLA-B*, *HLA-DQA1*, *HLA-DQB1*, *NOTCH4*	2.75 × 10^−4^
		***HLA-A***, ***HLA-DRA***	
IL-4 Signaling*	0.054	*HLA-B*, *HLA-DQA1*, *HLA-DQB1*	6.03 × 10^−4^
		***HLA-A***, ***HLA-DRA***	
Caveolar-mediated Endocytosis Signaling	0.053	*FLOT1*, *HLA-B*, *HLA-A*, *HLA-C*	2.24 × 10^−3^
Atherosclerosis Signaling	0.038	*APOC1*, *APOE*, *APOM*, *PLA2G12A*, ***MMP9***	2.75 × 10^−3^
Glucocorticoid Receptor Signaling*	0.019	*HLA-B*, *HLA-DQA1*, *HLA-DQB1*, *NDUFS7*, *PLA2G12A*	3.72 × 10^−3^
		***HLA-A***, ***HLA-C***, ***HLA-DRA***, ***MMP9***, ***POLR2E***, ***TGFBR1***	
Role of *OCT4* in Mammalian Embryonic	0.065	*CASP6*, *POU5F1*, ***REST***	4.57 × 10^−3^
Stem Cell Pluripotency*			
Phagosome Maturation*	0.031	*HLA-B*, *HLA-A*, *HLA-C*, *HLA-DRA*, *TUBB*	6.31 × 10^−3^

• Pathways with * are enriched only when both marginally strong and weak genes were included.

• Ratio = the proportion of candidate genes in the enriched pathway.

• Genes in bold typeface are marginally weak genes.

All nine canonical pathways enriched by both gene lists had been reported in the AMD literature, including the complement system (Anderson et al., 2010; Ishikawa et al., 2016; Potilinski et al., 2021), the antigen presentation (Ambati et al., 2013; Potilinski et al., 2021), the LXR/RXR activation (Mukwaya et al., 2018; Choudhary et al., 2020) and the FXR/RXR activation (Kurji et al., 2010) pathways. These results agree with the current knowledge that AMD is associated with inflammatory response, which requires the accumulation of various immune cells and cytokines in the eye (Tan et al., 2020). Additionally, leading-edge gene *THOP1* in the Alzheimer’s disease pathway, which is significantly enriched, is known to have a neuroprotective role. The elevated expression of thimet oligopeptidase (*THOP1*) is a neuroprotective response to amyloid-*β* (*Aβ*), a major component of Alzheimer’s disease plaques that may cause increased neuronal death (Pollio et al., 2008). This is consistent with the fact that *Aβ* also presents in the drusen of AMD patients (Pollio et al., 2008). Multiple studies indicated that atherosclerosis is a risk factor for AMD (Vingerling et al., 1995; van Leeuwen et al., 2003; Logue et al., 2014). The atherosclerosis signaling pathway was significantly enriched in our study and other studies (Fritsche et al., 2013; Zhang and Zhou, 2020). This supported the hypothesis that AMD and atherosclerosis may share common pathogenic pathways (Chau et al., 2008).

For the list with marginally weak genes, nine more canonical pathways were significantly enriched, including those involved in immune responses and inflammatory activities, in crosstalk between dendritic cells, Th1 and Th2 activation, and natural killer cells IL-4 Signaling. The neuroinflammation signaling pathway became significantly enriched by adding the marginally weak genes. Leading-edge genes in this pathway include *HLA-A*, *HLA-C*, *HLA-DRA*, *MMP9*, and *TGFBR1*. This finding is in line with earlier research that suggested neuroinflammation is involved in late-stage AMD (Buschini et al., 2011; Racic et al., 2021). By incorporating the weak genes *CSNK2B*, *HLA-A*, *HLA-C*, and *HLA-DRA*, the PD-1, PD-L1 cancer immunotherapy pathway was significantly enriched. This may be due to the fact that programmed death receptor-1 (PD-1) and its ligand PD-L1 play critical roles in the immune response and autoimmunity beyond cancer (Qin et al., 2019), including preserving the retinal immunosuppressive micro-environment (Chen et al., 2009). Marginally weak genes *HLA-A*, *HLA-C*, *HLA-DRA*, *MMP9*, *POLR2E*, *TGFBR1* were responsible for the enrichment of the glucocorticoid receptor signaling pathway. Glucocorticoid receptor is associated with anti-inflammatory properties and may play a crucial role in late-stage AMD progression (Jin et al., 2017). A synthetic glucocorticoid called triamcinolone is used to treat people with CNV-type AMD, according to Becerra et al. (2011). With marginally weak gene *REST*, the Role of *OCT4* in mammalian embryonic stem cell pluripotency pathway can be validated. *OCT4*, also known as *Pou5f1*, is important for maintaining and regaining stem cell pluripotency. According to a recent study, human embryonic stem cells (hESCs) can encourage the retro-differentiation of Müller cells in the retina into retinal progenitor cells by regulating the expression of *OCT4* in these cells (Ke et al., 2021). This also indicates that stem cells may provide a new option for treating AMD (Parameswaran and Krishnakumar, 2017; Ke et al., 2021). The enriched phagosome maturation pathway is in concordance with the facts that efficient RPE phagosome maturation is crucial for retinal health and homeostasis, and that the delay in maturation is related to the accumulation of debris in the RPE and AMD (Gordiyenko et al., 2010; Wavre-Shapton et al., 2013; Kwon and Freeman, 2020).

We also conducted a gene set enrichment analysis (GSEA) using the curated gene sets in Molecular Signature Database (MSigDB v7.5.1 c2). Multiple gene sets linked to AMD-related phenotype changes or biological processes were enriched. They include extracellular matrix organization, complement system, lipid metabolism, immune, and inflammation responses (Supplementary Table S6). Marginally weak genes (*REEP6*, *HLA-A*, *TGFBR1*, *POLDIP2*, *MMP9*, and *VTN*) are confirmed as leading edge genes within the AMD-related gene sets, including “GOBP: activation of the immune response,” “GOCC: photoreceptor inner segment,” “HP: choroidal neovasularization,” “HP: macular degeneration” among others. These serve to further illustrate the role that marginally weak genes have significant co-regulatory effects, even no significant differentiating effects, in the onset and course of the disease. Identification of these weak signals could provide new insights into a better understanding of the underlying disease mechanisms.

### 4.4 Prediction model based on selected SNPs

For prediction, we applied a five-fold cross-validation, where the whole dataset was partitioned into five folds of sub-datasets with similar sample sizes. In each round of cross-validation, we aggregated four folds of data into a training set for model training. We used the remaining fold as the test set for AMD status prediction. Through the five rounds of cross-validations, each sample in the original dataset would have been predicted once. Prediction performance was evaluated by comparing the predicted labels to the observed ones.

Our method, which takes into account both weak and strong signals, has a prediction accuracy of 0.769 with an area under the receiver operating characteristic curve (AUC) of 0.768. With only the 4,679 marginally strong SNPs, it achieves a prediction accuracy of 0.732 and an AUC of 0.730. This demonstrated that marginally weak signals could help to improve the prediction performance.

## 5 Discussion

Our study employed a novel machine-learning method to detect marginally weak variants in a GWAS. By exploring and integrating the genome-wide LD spectrum into GWAS, we were able to detect novel susceptibility genes for AMD and explain more disease variability. The previously identified genes like *BTBD16*, *C3*, *CFH*, *CFHR3*, *HTARA1*) were confirmed in our study. Novel AMD-related genes *DENND1B*, *PLK5*, *ARHGAP45*, and *BAG6* were detected as weak signals. More AMD-related biological pathways were enriched when marginally weak signals were considered.

For the effect size estimates of the selected SNPs, compared to the marginal approaches, the estimates from our approach would be closer to their oracle estimates, which are the estimates from fitting a joint model assuming one knew prior to the analysis the subset of true causal SNPs (Li et al., 2019b). It is possible that a marginally strong SNP turns out to have a weak joint effect. Such SNPs would be false positive findings in conventional GWA studies. Our approach diminishes such cases.

In our approach, each weak signal detected must have a high-LD path linking it to some marginally strong SNPs. It is cognizant that other than linking to strong signals, some weak signals work together by themselves as a group to exude their overall predictive effect on the outcomes, such as a group of rare variants (Li and Leal, 2008). Our approach would not detect such groups of weak signals. It is promising to combine our approach with the rare variant grouping methods in GWA studies. We will investigate this in future studies.

Our weak variants detection method can be directly applied to GWA studies with other types of trait variables: continuous, ordinal, counting, and survival outcomes. We envision its wide range of applications.

## Data Availability

Publicly available datasets were analyzed in this study. Genotypes and phenotypes for the samples analyzed in this study can be accessed at https://www.ncbi.nlm.nih.gov/projects/gap/cgi-bin/study.cgi?study_id=phs001039.v1.p1. R codes for the analysis pipeline can be downloaded at https://github.com/XuepingZhou/mLDA_Pipeline.
