# Supplementary material for: Predicting late-stage age-related macular degeneration by integrating marginally weak SNPs in GWA studies
Source: Front Genet. 2023 Mar 30;14:1075824. doi: 10.3389/fgene.2023.1075824 (PMC10101437; doi:10.3389/fgene.2023.1075824)
Supplement: Supplementary file 1 [file Table1.PDF]

Supplementary Material

Table S1. 63 candidate genes mapped by both marginally strong and marginally weak SNPs.

Chromosome	# of genes	Gene
1	8	*ASPM, CFH, CFHR3, CFHR4, CRB1, DENND1B, F13B, ZBTB4*
3	1	*COL8A1*
4	2	*CFI, MCUB*
6	32	*AIF1, APOM, ATAT1, BAG6, C6orf47, CCHCR1, GPANK1, GPSM3, HCG27, HLA-DQB1, HLA-DQB1-AS1, LINC00243, LINC02571, LSM2, MICB, MSH5, MSH5-SAPCD1, MUC21, MUC22, POU5F1, PRRC2A, PSORS1C1, PSORS1C3, SFTA2, SLC44A4, SNHG32, TCF19, TNXB, TSBP1, TSBP1-AS1, VARS1, VWA7*
7	2	*KMT2E, SRPK2*
8	1	*LOC389641*
10	4	*ARHGAP21, BTBD16, DMBT1, TACC2*
14	1	*RAD51B*
17	2	*NPLOC4, TMEM97*
19	8	*ABHD17A, CSNK1G2, EXOC3L2, FSTL3, PCSK4, PRSS57, REXO1, SHC2*
22	2	*BAIAP2L2, SLC16A8*

[b]

Table S2. 99 candidate genes uniquely mapped by marginally strong SNPs.

Chromosome	# of genes	Gene
1	4	*CFHR1, CFHR2, CFHR5, KCNT2*
2	2	*COL4A3, MFF-DT*
3	1	*ADAMTS9-AS2*
4	3	*CASP6, IGFBP7, PLA2G12A*
6	34	*AIF1, APOM, ATAT1, BAG6, C6orf47, CCHCR1, GPANK1, GPSM3, HCG27, HLA-DQB1, HLA-DQB1-AS1, HSPA1A, HSPA1L, LINC00243, LINC02571, LSM2, MICB*, MSH5, *MSH5-SAPCD1, MUC21, MUC22, POU5F1, PRRC2A, PSORS1C1, PSORS1C3, SFTA2, SLC44A4, SNHG32, TCF19, TNXB, TSBP1, TSBP1-AS1,VARS1, VWA7*
7	11	*C7orf61, MEPCE, NYAP1, PILRA, PILRB, PLEKHA1, PVRIG2P, STAG3L5P, STAG3L5P-PVRIG2P-PILRB, TSC22D4, ZCWPW1*
9	3	*ABCA1, TRPM3, VAV2*
10	5	*ARMS2, C10orf120, DMBT1L1, HTRA1, PLEKHA1*
12	2	*RDH5, BLOC1S1-RDH5*
13	1	*B3GLCT*
14	1	*RDH12*
15	2	*LIPC, LIPC-AS1*
16	1	*CETP*
19	28	*ABCA7, APOC1, APOE, ARHGAP45, ARID3A, ATP8B3, C3, CFD, CNN2, DAZAP1, ELANE, GAMT, GPR108, GRIN3B, KISS1R, KLF16, MED16, NDUFS7, NECTIN2, PLK5, POLR2E, PRTN3, PWWP3A, R3HDM4, TMEM259, TOMM40, TPGS1, TRIP10*
22	1	*SYN3*

[b]

Table S3. 35 candidate genes uniquely mapped by marginally weak SNPs.

Chromosome	# of genes	Gene
4	1	*REST*
6	21	*ABCF1-DT, ATP6V1G2-DDX39B, CSNK2B, DDAH2, DDX39B, FLOT1, GNL1, HCG20, HCP5, HLA-A, HLA-C, HLA-DRA, LINC01149, MDC1,MICB-DT, SRPK2, TFAP2B, TRIM31, TUBB, VARS2, ZFP57*
7	1	*PMS2P1*
9	1	*TGFBR1*
17	6	*KRT18P55, POLDIP2, SARM1, TNFAIP1, TSPAN10, VTN*
19	2	*REEP6, WDR18*
20	3	*MMP9, SLC12A5, SLC12A5-AS1*

Table S4. Statistically significant marginally strong SNPs based on permutation tests and associated genes (extended version).

Ref SNP	CHR:POS	Gene	AMD-related Literature
rs6695321	1:196675861	*CFH*	(Guenther et al., 2020; Tian et al., 2012)*
rs7524776	1:196623337	*CFH*	(Budzinskaia et al., 2011)*
rs1853881	1:196915745	*CFHR2*	(Kubista et al., 2011)
rs59745661	1:196955502	*CFHR5*	(Narendra et al., 2009)
rs76622629	1:196942623	*CFHR5*	as above
rs201084593	1:197316970	*CRB1*	(Fletcher et al., 2014)
rs4915551	1:197508901	*DENND1B*	
rs4388642	1:196494547	*KCNT2*	(Cipriani et al., 2020)
rs4658039	1:196513323	*KCNT2*	as above
rs16840224	1:196569681	*KCNT2*	as above
rs144106834	1:196277290	*KCNT2*	as above
rs35078847	1:196303302	*KCNT2*	as above
rs10429910	1:197141253	*ZBTB41*	(Zhang et al., 2008)
rs150519105	1:197155951	*ZBTB41*	as above
rs186543187	1:197133991	*ZBTB41*	as above
rs61578883	1:196820514	None	
rs78558010	1:196982580	None	
rs79497719	1:196783242	None	
rs199921106	1:196820511	None	
rs200519349	1:197044393	None	
rs4865172	4:57907587	*IGFBP7*	(Arakawa et al., 2011)
rs3132451	6:31582025	*AIF1*	(Wolf et al., 2022)
rs201943238	6:32314956	*TSBP1, TSBP1-AS1*	(Strunz, 2021)
rs1043618	6:31783507	*HSPA1A*	(Liang et al., 2022)
rs444921	6:31932177	*SKIV2L*	(Kopplin et al., 2010)
rs406658	6:31996524	*C4B*	(Grassmann et al., 2016)
rs6906021	6:32626311	*HLA-DQB1-AS1*	(Jorgenson et al., 2016)
rs9391844	6:31319119	None	
rs9391845	6:31319120	None	
rs5788544	10:124091856	*BTBD16*	(Strunz et al., 2020)
rs3887220	10:124091572	*BTBD16*	as above
rs10559219	10:124040080	*BTBD16*	as above
rs11200554	10:124085243	*BTBD16*	as above
rs2104176	10:124045854	*BTBD16*	as above
rs2981804	10:124333656	*DMBT1*	(Polley et al., 2016)
rs4237541	10:124237947	*HTRA1*	(DeWan et al., 2006)
rs4547034	10:124238057	*HTRA1*	
rs2300431	10:124242817	*HTRA1*	(Gibbs et al., 2008)*
rs7093894	10:124234880	*HTRA1*	(Goto et al., 2009)*
rs2239586	10:124249235	*HTRA1*	(Tam et al., 2008)*
rs11200645	10:124238153	*HTRA1*	as above
rs202015126	10:124150339	*PLEKHA1*	(Yu et al., 2013)
rs72631113	10:124213449	*ARMS2* †	(Vavvas et al., 2018)
rs56029211	10:124127990	None	
rs7068411	10:124202878	None	
rs4430444	10:124295405	None	
rs7085821	10:124102664	None	
rs2038596	10:124114152	None	
rs7920206	10:124099644	None	
rs3019478	10:124315798	None	
rs7091160	10:124103876	None	
rs6857	19:45392254	*NECTIN2/PVRL2*	(Holliday et al., 2013)*
rs265285	19:1534676	*PLK5*	
rs265284	19:1534685	*PLK5*	
rs2074453	19:1080189	*ARHGAP45/HMHA1*	

CHR:POS stands for chromosome position, and is based on Genome Reference Consortium Human Build 37. SNP with † is a 2kb upstream variant of the associated gene. Literature with * is SNP level AMD-related study.

Table S5. Enriched canonical pathways of candidate genes mapped by marginally strong SNPs using Ingenuity Pathway Analysis.

**Ingenuity Canonical Pathway**	**Ratio**	**Pathway Genes**	**p value**
Complement System	0.162	*C2,C3,C4A/C4B,CFD,CFH,CFI*	9.33E-08
LXR/RXR Activation	0.057	*ABCA1,APOC1,APOE,APOM,C3,C4A/C4B,CETP*	1.10E-05
FXR/RXR Activation	0.056	*APOC1,APOE,APOM,C3,C4A/C4B,CETP,LIPC*	1.29E-05
Neuroprotective Role of THOP1 in Alzheimer's Disease	0.042	*CFD,HLA-B,HTRA1,PRSS57,PRTN3*	8.71E-04
Antigen Presentation Pathway	0.077	*HLA-B,HLA-DQA1,HLA-DQB1*	1.74E-03
B Cell Development	0.068	*HLA-B,HLA-DQA1,HLA-DQB1*	2.45E-03
Th1 Pathway	0.033	*HLA-B,HLA-DQA1,HLA-DQB1,NOTCH4*	6.76E-03
Atherosclerosis Signaling	0.031	*APOC1,APOE,APOM,PLA2G12A*	8.71E-03
Th2 Pathway	0.029	*HLA-B,HLA-DQA1,HLA-DQB1,NOTCH4*	1.00E-02

Ratio is the number of candidate genes that maps to the pathway divided by the total number of genes in the same pathway

Table S6. Selected enriched gene sets using gene set enrichment analysis (GSEA).

Gene Set in the Molecule Signatures Database	p adjusted	NES	leading edge gene
**Complement system**			
REACTOME_COMPLEMENT_CASCADE	1.23E-10	-3.57E+00	*CFHR1, CFHR2, CFHR5, C4B, CFD, ELANE*
HP_ABNORMALITY_OF_COMPLEMENT_SYSTEM	6.69E-07	-3.22E+00	*CFHR1, CFHR2, C4B, CFD*
HP_DECREASED_SERUM_COMPLEMENT_FACTOR_I	1.46E-05	-2.55E+00	CFHR1
WP_COMPLEMENT_SYSTEM	2.63E-05	-2.87E+00	*CFHR2, CFD, ELANE*
HP_DECREASED_SERUM_COMPLEMENT_FACTOR_B	5.19E-05	-2.28E+00	CFHR1
GOBP_COMPLEMENT_ACTIVATION	6.46E-05	-2.85E+00	*CFHR1, CFHR5, C4B, CFD*
HP_DECREASED_SERUM_COMPLEMENT_C4	4.16E-04	-2.47E+00	*CFHR2, C4B*
HP_DECREASED_SERUM_COMPLEMENT_C3	2.04E-03	-2.62E+00	*CFHR1, CFHR2*
GOBP_COMPLEMENT_ACTIVATION_ALTERNATIVE_PATHWAY	4.51E-03	-2.40E+00	*CFHR5, CFD*
**Extracellular matrix organization**			
GOBP_NEGATIVE_REGULATION_OF_PROTEIN_BINDING	2.04E-03	-2.51E+00	*CFHR1, CFHR2, CFHR5*
GOBP_ADHERENS_JUNCTION_MAINTENANCE	3.04E-03	2.20E+00	
GOBP_REGULATION_OF_PROTEIN_BINDING	3.47E-03	-2.19E+00	*CFHR1, CFHR2, CFHR5, APOE*
GOMF_PROTEOGLYCAN_BINDING	4.96E-03	-2.46E+00	APOE
NABA_ECM_REGULATORS	7.62E-03	2.02E+00	HTRA1
REACTOME_EXTRACELLULAR_MATRIX_ORGANIZATION	2.15E-02	1.83E+00	DDR1, *HTRA1*
GOCC_PERICILIARY_MEMBRANE_COMPARTMENT	3.01E-02	1.84E+00	**TGFBR1, POLDIP2, MMP9**
GOMF_GLYCOSAMINOGLYCAN_BINDING	4.31E-02	-1.83E+00	LIPC, *APOE, ELANE*
GOBP_NEGATIVE_REGULATION_OF_BINDING	4.49E-02	-1.98E+00	*CFHR1, CFHR2, CFHR5*
**Immune and inflammation**			
GOBP_ACTIVATION_OF_IMMUNE_RESPONSE	3.30E-05	-2.30E+00	*CFHR1, CFHR5, CASP6, C4B, PLEKHA1, CFD,* ***VTN***
GOBP_POSITIVE_REGULATION_OF_IMMUNE_RESPONSE	1.21E-04	-2.03E+00	*CFHR1, CFHR5, CASP6, C4B, PLEKHA1, CFD, ELANE,* ***HLA-A***

Gene in **Bold** denote marginally weak signals.

Table S7. Selected enriched gene sets using gene set enrichment analysis (Continued).

Gene Set in the Molecule Signatures Database	p adjusted	NES	leading edge gene
**AMD Related physiological changes**			
HP_DRUSEN	1.37E-07	-3.31E+00	*CFHR1, CFHR2, APOE*
HP_YELLOW_WHITE_LESIONS_OF_THE_RETINA	2.56E-06	-3.17E+00	*CFHR1, CFHR2, APOE*
HP_ABNORMAL_MORPHOLOGY_OF_THE_CHOROIDAL_VASCULATURE	3.81E-06	-3.06E+00	*CFHR1, APOE,**HLA-A***
HP_YELLOW_WHITE_LESIONS_OF_THE_MACULA	5.54E-06	-2.89E+00	*CFHR1, APOE*
HP_ABNORMALITY_OF_MACULAR_PIGMENTATION	1.43E-05	-3.04E+00	*CFHR1, APOE*
HP_HEMORRHAGE_OF_THE_EYE	2.63E-05	-3.14E+00	*CFHR1, APOE*
HP_MACULAR_HEMORRHAGE	3.15E-05	-2.53E+00	*CFHR1, APOE*
HP_CHOROIDAL_NEOVASCULARIZATION	3.75E-05	-3.03E+00	*CFHR1, APOE,**HLA-A***
HP_GEOGRAPHIC_ATROPHY	9.78E-05	-2.38E+00	*CFHR1, APOE*
HP_VISUAL_LOSS	4.50E-04	-2.28E+00	*CFHR1, CFHR2, APOE,**HLA-A***
HP_ABNORMALITY_OF_FOVEAL_PIGMENTATION	4.82E-04	-2.46E+00	*CFHR1, APOE*
HP_ABNORMAL_CHOROID_MORPHOLOGY	8.55E-04	-2.37E+00	*CFHR1, CFHR2, APOE,**HLA-A***
GOCC_PHOTORECEPTOR_INNER_SEGMENT	3.18E-03	2.72E+00	*ARMS2, RDH12,**REEP6***
HP_MACULAR_DEGENERATION	3.57E-03	-2.56E+00	*CFHR1, APOE,**HLA-A***
HP_ABNORMAL_MACULAR_MORPHOLOGY	5.95E-03	-2.17E+00	*CFHR1, APOE,**HLA-A***
HP_ABNORMAL_CHORIORETINAL_MORPHOLOGY	7.77E-03	-2.21E+00	*CFHR2,**HLA-A***
HP_ABNORMAL_FOVEAL_MORPHOLOGY	1.05E-02	-2.35E+00	*CFHR1, APOE*
HP_PROGRESSIVE_VISUAL_LOSS	1.05E-02	-2.46E+00	*CFHR1, APOE*
**Lipid metabolism**			
GOMF_PHOSPHOLIPID_BINDING	5.54E-03	1.84E+00	
WP_STATIN_INHIBITION_OF_CHOLESTEROL_PRODUCTION	1.27E-02	-2.30E+00	*ABCA1, LIPC, CETP, APOC1, APOE*
HP_ABNORMAL_CIRCULATING_LIPID_CONCENTRATION	1.57E-02	-1.94E+00	*CFHR1, ABCA1, LIPC, CETP, APOE*
GOBP_PHOSPHATIDYLCHOLINE_CATABOLIC_PROCESS	2.84E-02	-2.26E+00	LIPC, *APOC1*
GOBP_LOW_DENSITY_LIPOPROTEIN_PARTICLE_REMODELING	3.14E-02	-2.25E+00	LIPC, *CETP, APOE,**HLA-A***
GOBP_PROTEIN_LIPID_COMPLEX_SUBUNIT_ORGANIZATION	3.60E-02	-2.13E+00	*ABCA1, LIPC, CETP, ABCA7, APOC1, APOE*
GOBP_PHOSPHOLIPID_TRANSPORT	4.08E-02	-2.12E+00	*ABCA1, CETP, ABCA7, APOC1, APOE*
GOBP_TRIGLYCERIDE_RICH_LIPOPROTEIN_PARTICLE_CLEARANCE	4.18E-02	-2.08E+00	LIPC, *APOC1, APOE*
**Multiple Process**			
KEGG_MAPK_SIGNALING_PATHWAY	3.58E-02	-1.81E+00	*PLA2G12A*

Gene in **Bold** denote marginally weak signals.

REFERENCES

Arakawa, S., Takahashi, A., Ashikawa, K., Hosono, N., Aoi, T., Yasuda, M., et al. (2011). Genome-wide association study identifies two susceptibility loci for exudative age-related macular degeneration in the japanese population. *Nature genetics* 43, 1001–1004

Budzinskaia, M., Pogoda, T., Generozov, É., Chikun, E., Shchegoleva, I., Kazarian, É., et al. (2011). Influence of genetic mutations on clinical presentation of subretinal neovascularization. report 1: The impact of cfh and il-8 genes polymorphism. *Vestnik Oftalmologii* 127, 3–8

Cipriani, V., Lorés-Motta, L., He, F., Fathalla, D., Tilakaratna, V., McHarg, S., et al. (2020). Increased circulating levels of factor h-related protein 4 are strongly associated with age-related macular degeneration. *Nature communications* 11, 1–15

DeWan, A., Liu, M., Hartman, S., Zhang, S. S.-M., Liu, D. T., Zhao, C., et al. (2006). Htra1 promoter polymorphism in wet age-related macular degeneration. *Science* 314, 989–992

Fletcher, E. L., Jobling, A. I., Greferath, U., Mills, S. A., Waugh, M., Ho, T., et al. (2014). Studying age-related macular degeneration using animal models. *Optometry and Vision Science* 91, 878

Gibbs, D., Yang, Z., Constantine, R., Ma, X., Camp, N. J., Yang, X., et al. (2008). Further mapping of 10q26 supports strong association of htra1 polymorphisms with age-related macular degeneration. *Vision research* 48, 685–689

Goto, A., Akahori, M., Okamoto, H., Minami, M., Terauchi, N., Haruhata, Y., et al. (2009). Genetic analysis of typical wet-type age-related macular degeneration and polypoidal choroidal vasculopathy in japanese population. *Journal of ocular biology, diseases, and informatics* 2, 164–175

Grassmann, F., Cantsilieris, S., Schulz-Kuhnt, A.-S., White, S. J., Richardson, A. J., Hewitt, A. W., et al. (2016). Multiallelic copy number variation in the complement component 4a (c4a) gene is associated with late-stage age-related macular degeneration (amd). *Journal of neuroinflammation* 13, 1–9

Guenther, F., Brandl, C., Winkler, T. W., Wanner, V., Stark, K., Kuechenhoff, H., et al. (2020). Chances and challenges of machine learning-based disease classification in genetic association studies illustrated on age-related macular degeneration. *Genetic Epidemiology* 44, 759–777

Holliday, E. G., Smith, A. V., Cornes, B. K., Buitendijk, G. H., Jensen, R. A., Sim, X., et al. (2013). Insights into the genetic architecture of early stage age-related macular degeneration: a genome-wide association study meta-analysis. *PloS one* 8, e53830

Jorgenson, E., Melles, R. B., Hoffmann, T. J., Jia, X., Sakoda, L. C., Kvale, M. N., et al. (2016). Common coding variants in the hla-dqb1 region confer susceptibility to age-related macular degeneration. *European Journal of Human Genetics* 24, 1049–1055

Kopplin, L. J., Igo, R., Wang, Y., Sivakumaran, T. A., Hagstrom, S. A., Peachey, N. S., et al. (2010). Genome-wide association identifies skiv2l and myrip as protective factors for age-related macular degeneration. *Genes & Immunity* 11, 609–621

Kubista, K. E., Tosakulwong, N., Wu, Y., Ryu, E., Roeder, J. L., Hecker, L. A., et al. (2011). Copy number variation in the complement factor h-related genes and age-related macular degeneration. *Molecular vision* 17, 2080

Liang, G., Ma, W., Luo, Y., Yin, J., Hao, L., and Zhong, J. (2022). Identification of differentially expressed and methylated genes and construction of a co-expression network in age-related macular degeneration. *Annals of Translational Medicine* 10

Narendra, U., Pauer, G. J., and Hagstrom, S. A. (2009). Genetic analysis of complement factor h related 5, cfhr5, in patients with age-related macular degeneration. *Molecular vision* 15, 731

Polley, S., Cipriani, V., Khan, J. C., Shahid, H., Moore, A. T., Yates, J. R., et al. (2016). Analysis of copy number variation at dmbt1 and age-related macular degeneration. *BMC medical genetics* 17, 1–8

Strunz, T. (2021). *Genetic Variants with Significant Association to Age-Related Macular Degeneration (AMD) and their Role in the Regulation of Gene Expression*. Ph.D. thesis

Strunz, T., Kiel, C., Sauerbeck, B. L., and Weber, B. H. (2020). Learning from fifteen years of genome-wide association studies in age-related macular degeneration. *Cells* 9, 2267

Tam, P. O., Ng, T. K., Liu, D. T., Chan, W. M., Chiang, S. W., Chen, L. J., et al. (2008). Htra1 variants in exudative age-related macular degeneration and interactions with smoking and cfh. *Investigative ophthalmology & visual science* 49, 2357–2365

Tian, J., Yu, W., Qin, X., Fang, K., Chen, Q., Hou, J., et al. (2012). Association of genetic polymorphisms and age-related macular degeneration in chinese population. *Investigative ophthalmology & visual science* 53, 4262–4269

Vavvas, D. G., Small, K. W., Awh, C. C., Zanke, B. W., Tibshirani, R. J., and Kustra, R. (2018). Cfh and arms2 genetic risk determines progression to neovascular age-related macular degeneration after antioxidant and zinc supplementation. *Proceedings of the National Academy of Sciences* 115, E696–E704

Wolf, J., Schlecht, A., Rosmus, D.-D., Boneva, S., Agostini, H., Schlunck, G., et al. (2022). Comparative transcriptome analysis of human and murine choroidal neovascularization identifies fibroblast growth factor inducible-14 as phylogenetically conserved mediator of neovascular age-related macular degeneration. *Biochimica et Biophysica Acta (BBA)-Molecular Basis of Disease* , 166340

Yu, W., Dong, S., Zhao, C., Wang, H., Dai, F., and Yang, J. (2013). Cumulative association between age-related macular degeneration and less studied genetic variants in plekha1/arms2/htra1: a meta and gene-cluster analysis. *Molecular biology reports* 40, 5551–5561

Zhang, H., Morrison, M. A., DeWan, A., Adams, S., Andreoli, M., Huynh, N., et al. (2008). The nei/ncbi dbgap database: genotypes and haplotypes that may specifically predispose to risk of neovascular age-related macular degeneration. *BMC medical genetics* 9, 1–10
